# Parity of Indigenous and Non-Indigenous Women in Brazil: Does the Reported Number of Children Born Depend upon Who Answers National Census Questions?

**DOI:** 10.1371/journal.pone.0123826

**Published:** 2015-04-14

**Authors:** Ricardo Ventura Santos, João Luiz Bastos, Oswaldo Gonçalves Cruz, Luciene Aparecida Ferreira de Barros Longo, Nancy May Flowers, Nilza de Oliveira Martins Pereira

**Affiliations:** 1 Escola Nacional de Saúde Pública, Fundação Oswaldo Cruz, Rio de Janeiro, RJ, Brazil; 2 Departamento de Saúde Pública, Universidade Federal de Santa Catarina, Campus Universitário Trindade, Florianópolis, SC, Brazil; 3 Programa de Computação Científica, Fundação Oswaldo Cruz, Rio de Janeiro, RJ, Brazil; 4 Instituto Brasileiro de Geografia e Estatística, Unidade Estadual de Minas Gerais, Belo Horizonte, MG, Brazil; 5 Hunter College, City University of New York, New York, NY, United States of America; 6 Instituto Brasileiro de Geografia e Estatística, Diretoria de Pesquisas, Rio de Janeiro, RJ, Brazil; Örebro University, SWEDEN

## Abstract

Taking parity as the main analytic variable, the objective of this study is to investigate whether the patterns of response to national census questions in Brazil differ when Indigenous and non-Indigenous women are compared, taking into consideration whether the information was provided by the women directly or by a proxy respondent (another household member or a non-resident). We use data on children ever born to Indigenous and non-Indigenous women from two Brazilian regions, the Northeast and the North. Data on the number of household members, total household rooms, interviewee’s color/race, educational attainment, age, parity, and type of respondent were obtained from the 2010 Brazilian census. The relation between color/race and reported parity, as well as the impact of the type of respondent on this association were assessed with the Zero-inflated Negative Binomial regression, stratified by region (North and Northeast) and urban/rural status. Just over half of census interviewees answered directly the census questions (51.2% in the North and 54.4% in the Northeast). Indigenous women in the North region had the highest percentage of interviews carried out with a non-resident (12.7% total; 15.0% and 3.0% in rural and urban areas, respectively). Regardless of color/race, parity means were considerably higher when the question was answered by the woman directly (93.5%-101.4% and 15.6%-21.7% higher, compared co-resident and non-resident based answers, respectively). Parity underreporting was particularly strong in Indigenous women living in the rural North (16.0% less in comparison to White women). Proxy respondents tend to underestimate the count of children, particularly among Indigenous women from the North. The implementation of certain methodological alternatives in the Brazilian national censuses, such as the selection and training of census takers to work specifically in Indigenous territories, might be a productive means to improve data collection.

## Introduction

Due to distinct historical trajectories, different forms of classification, and the lack of accurate statistics, it is difficult to estimate the number of Indigenous peoples across the globe [1–3]. However, there has been a growing concern with demographic and health data on these ethnic groups, who tend to have lower living conditions compared to the surrounding national societies [2–5]. In order to describe the living conditions and to assess the levels of inequity in health and demographic conditions, international agencies have been encouraging countries worldwide to include questions pertaining to the socio-demographic conditions of Indigenous peoples in their national census surveys [2, 3].

It is estimated that there are over 400 different Indigenous groups in Latin America and the Caribbean, with a population of nearly 50 million [6, 7]. While some countries, including Bolivia, Ecuador, Guatemala and Peru, have over 40% of their populations constituted by Indigenous peoples, in others, such as Argentina, Brazil, Costa Rica and Uruguay, the percentages are below 1% [7]. As in other regions of the world, Indigenous peoples in Latin America present lower living standards, lower educational attainment, higher mortality levels and other negative social and health indicators compared to the wider national societies [2, 6–9].

In recent decades, the majority of Latin American countries have included, and actually expanded, the questions pertaining to Indigenous peoples in their national censuses [10, 11]. The number of questions, as well as their specific content, vary from country to country, though a common denominator has been the attempt to collect data on the size of the Indigenous populations and on their ethnic affiliations. The Brazilian case is illustrative in this regard. Starting in 1991, the category “Indigenous” was included as one of the options in the color/race question of the Brazilian national censuses. In the 2010 Brazilian census, whenever the person chose “Indigenous”, he/she was also asked about ethnic affiliation and native languages spoken in the household [12–14].

It is unquestionable that the inclusion of demographic characteristics of Indigenous peoples in nationwide censuses in Latin America and elsewhere is a major step toward better documenting their living conditions and thereby implementing and evaluating the impacts of a broad range of public policies [3, 7, 10, 11]. However, it is important to recognize that, to a varying extent, social and cultural characteristics of Indigenous societies in conjunction with specific methodological procedures employed in the national census may influence the way Indigenous interviewees respond to questions posed by census takers [1]. For instance, in the case of Brazil, many Indigenous communities have few or no speakers of Portuguese, which is the language used in the census [13]. Other important cultural aspects that may affect census reporting are Indigenous counting systems, which in most groups that retain their native languages is different from the decimal system commonly used in demographic analyses, and Indigenous systems of age reckoning, which often are not based on birthdate [15]. The influence of these factors should be considered when looking at census data for Indigenous peoples.

Focusing on reported parity as the main analytic variable, the objective of this study is to investigate whether the patterns of response to census questions differ when Indigenous and non-Indigenous women are compared, taking into consideration whether the information was provided by the women directly or by a proxy respondent (another household member or a non-resident). We use data on children ever born to Indigenous and non-Indigenous women from two Brazilian regions, the Northeast and the North. These regions were chosen because they differ markedly in the historical and anthropological characteristics of their Indigenous peoples, with a much larger number of Indigenous people speaking native languages in the North than the Northeast, among other contrasts. We expect that our analysis will shed light on factors that may have influenced the results of the Brazilian 2010 census, thus hopefully informing the planning and implementation of future census activities aimed at collecting data on Indigenous and other population segments. On a broader scale, our analysis provides useful information to rethink how data on Indigenous ethnic minorities, which are among the most marginalized in the world in terms of health and human rights, has been collected by national states.

## Materials and Methods

In Brazil, national demographic censuses have been carried out since the second half of the nineteenth century. Since 1940, the Brazilian Institute of Geography and Statistics (IBGE – http://www.ibge.gov.br) has been the responsible federal agency, and it has the task of carrying out a national census survey every ten years [16–18].

The 2010 Brazilian national census utilized two types of questionnaires. The basic questionnaire was applied to the universe of the Brazilian population, while the other, the sample questionnaire, only to the census sample. The basic questionnaire covered a limited range of household characteristics (mostly related to household composition and sanitation) and socio-demographic information for household members (including age, sex, color/race, literacy, and income). All questions from the basic questionnaire were also part of the sample questionnaire, which additionally included questions about occupation, fertility, migration, among others. Details of each 2010 census questionnaire may be found at http://censo2010.ibge.gov.br/ [19].

In each household, one person was interviewed (often an adult, identified as the household head) by the census taker, and he/she provided information regarding him/herself, as well as all other co-residents. As part of the 2010 census methodology, a non-resident of the household could also provide information regarding the household members. In order to identify these various respondents, the 2010 census database included a variable named “type of respondent” (“marca,” in Portuguese), with the following categories: (a) the information was provided directly by the person, that is, it was self-reported; (b) the information on a given household member was provided by a co-resident; and (c) the information on a given household member was provided by a non-resident (often, a domestic worker who happened to be at the house when the interview visit took place while no resident was present). Out of the nearly 190 million Brazilians surveyed, 4.2% had their answers provided by non-residents.

The 2010 Brazilian national census was the first in the country’s history to run the interviews with the aid of a Personal Digital Assistant, a mobile device that functions as an information manager, avoiding the use of paper-based questionnaires. In addition, differently from previous census editions, this was the first to include the color/race question in the basic questionnaire rather than only in the sample questionnaire. As an important advance in data collection, the 2010 census also collected data on Indigenous ethnic affiliation [13], Indigenous languages spoken in the household and whether the Indigenous person lived in federally recognized reserves. The color/race item divided the population into five discrete categories: white (“branca”), black (“preta”), yellow (“amarela,” in reference to those individuals of Asiatic origin, including Japanese, Chinese, Korean etc.), brown (“parda,” in reference to mestizos), and Indigenous [19]. Respondents could ignore this question, in which case they were assigned to a category labeled “unknown.” These individuals (<0.1% of the total population) were excluded from the present analysis.

Census takers who participated in the 2010 census did not receive specific training to conduct interviews in Indigenous communities [19]. However, the general training materials used by IBGE included some specific orientations aimed at better understanding Indigenous individuals and households, with a focus on housing patterns, religious practices, and local economies, given that these topics were addressed in part of the census questionnaires [19, 20]. In regions of the country with high concentrations of Indigenous lands, IBGE also made available to interviewers a pamphlet with general information about Indigenous peoples, prepared in cooperation with anthropologists and demographers affiliated with the Brazilian Association for Population Studies Working Group on Indigenous Demography (ABEP). In this case, the major aim was to sensitize interviewers to social and cultural patterns that they might face in Indigenous territories, from potentially encountering unclothed individuals to the expectation that they introduce themselves to village leaders prior to data collection.

For the purpose of this study, a subgroup of the 2010 census individual-level variables was converted into Stata format (Stata Corporation, College Station, Texas, USA), comprising information on: sample structure (including individual census tracts as primary sample units and contiguous groups of census tracts as strata), sampling weights, urban/rural status, number of household members, number of household rooms, interviewee’s color/race, educational attainment, marital status, and age. The main variable under analysis in the present study (parity, or the number of children ever born to a woman) was part of the variable set, and was defined as the count of children born alive up to July 2010, as reported to the census interviewers.

Analysis was restricted to females over 10 years of age living in the North and Northeast regions. These two regions were selected for comparison because their Indigenous peoples present some certain marked socio-historical contrasts. While Indigenous peoples of the Northeast were the first to suffer the impacts of the arrival of Europeans in the sixteenth century, those in the North region, which largely overlaps with the Amazon region, have had sustained interaction with non-Indigenous people much more recently, especially in the twentieth century [21, 22]. As a result of these complex and distinct historical trajectories, many more Indigenous groups in the North speak native languages, live in large Indigenous reserves, and maintain economic regimes based on traditional subsistence practices [21, 22]. Given the many centuries of non-Indian presence, Indigenous peoples living in the Northeast not only lost most of their traditional territories, but also cultural changes led to high rates of extinction of Indigenous languages. As an example of the different impacts of historical transformation in the two regions, while 55.2% of the self-declared Indigenous subjects in the North reported speaking an Indigenous language at home, this proportion was 13.6% in the Northeast [13]. Furthermore, the North region has the largest Indigenous population (342,836 individuals from a total of 896,917 in the whole country). The population of the Northeast includes an appreciable number of Indigenous people, 126,597 individuals, mostly living outside Indigenous areas [13]. In addition, the Northern region has the highest percentage (82.0%) of Indigenous groups in rural areas [13].

The first step of data analysis was the description, in both of the studied regions, of each color/race group according to urban/rural status, educational attainment, and age. Next, two important parameters for this study, that is, (1) frequency of interviews with each type of respondent and (2) parity, were estimated according to urban/rural status, color/race, age (in five-year cohorts up to 60+ years) and educational attainment (from illiterate up to complete high school, and unknown) for the North and Northeast regions separately. Parity was also estimated according to the type of respondent. Finally, the relation between color/race and parity, as well as the impact of the type of respondent on this association, were assessed in the context of Zero-inflated Negative Binomial (ZINB) regression models, stratified by region (North and Northeast) and urban/rural status. Details regarding the choice of the most appropriate regression model are available as S1 Information.

As a means of assessing whether the association between color/race and parity differed according to type of respondent, an interaction term between the variables color/race and type of respondent was added to the regression equations. Assessment of such an interaction followed the hypothesis that Indigenous women would be less likely than non-Indigenous women to respond directly to census interviewers due to higher rates of Portuguese non-fluency and culturally informed gender roles (for instance, men being more likely to interact with non-indigenous visitors). This would increase the likelihood that responses be reported by individuals, such as another household member or a non-resident, with less direct knowledge of a woman´s reproductive history. The Bayesian Information Criterion, Likelihood-ratio test, and Akaike Information Criterion indicated that models that included the interaction term consistently showed a better fit to the data (further information on model diagnostics is available as S2 Information.

Covariates in the models were age, educational attainment, number of household rooms (a count variable ranging from 1 to 30), and number of household members (a count variable ranging from 1 to 56). All independent variables were maintained in the adjusted regression models, irrespective of their impact on fit indices. As a means to ease interpretation, only coefficients for the count distribution are presented and discussed in this paper – coefficients estimated by the logistic part of the model are presented as S1 Table. The mean counts of children predicted by the models, with their respective 95% confidence intervals (95%CI), according to color/race and type of respondent, were also estimated, stratified by region and urban/rural status. All analyses took into account the complex sampling structure, as recommended by IBGE.

IBGE provides public-domain access to national census data. Consequently, and in accordance with current Brazilian guidelines for ethics in research with human subjects using secondary data (Conselho Nacional de Saúde/Brazilian National Health Council resolution no. 466/2012), there was no need for specific approval by an ethics and research committee.

## Results

The present analysis comprised 6,290,856 female respondents over 10 years of age in the North region of Brazil, and 22,817,444 in the Northeast (Table 1). The proportions of women classified as white, black, yellow, brown and Indigenous, respectively, in each region were: North – 24.0%, 6.2%, 1.3%, 66.9%, and 1.6%; Northeast – 29.6%, 9.4%, 1.4%, 59.2%, and 0.4%. Between 76.3% (brown) and 83.2% (yellow) of female residents in the North were located in urban areas; the exception was Indigenous women, whose place of residence was more likely to be in rural areas (75.7%). In contrast, in the Northeast the percentage of Indigenous women residing in urban areas was considerably higher (57.7%). The proportion of non-Indigenous women in the Northeast residing in urban areas ranged from 73.0% (brown) to 79.8% (white), slightly lower than in the North.

**Table 1:** Urban/rural status, educational attainment, and age, according to color/race, stratified by region.

Variables	Color/race – %									
	North					Northeast				
	White	Black	Yellow	Brown	Indigenous	White	Black	Yellow	Brown	Indigenous
**Urban/rural status**										
Urban	82.5	76.7	83.2	76.3	24.3	79.8	78.8	77.7	73.0	57.7
Rural	17.5	23.3	16.8	23.7	75.7	20.2	21.2	22.3	27.0	42.3
**Educational attainment**										
Illiterate/incomplete primary education	45.5	57.8	44.7	54.6	82.4	48.5	58.4	50.9	58.4	64.3
Complete primary education/ incomplete secondary education	17.0	16.2	19.2	17.5	8.7	15.6	15.2	17.1	16.1	15.2
Complete secondary education/ incomplete high school	29.7	20.5	28.1	22.4	7.2	25.4	22.0	25.2	20.6	16.4
Complete high school	10.1	5.0	7.1	4.8	1.2	10.0	3.9	6.2	4.4	3.7
Unknown	0.7	0.5	0.9	0.7	0.5	0.5	0.5	0.6	0.5	0.4
**Age (years)**										
10–14	12.3	11.2	11.1	14.4	19.2	10.4	9.1	10.0	12.1	13.2
15–19	12.3	11.3	13.0	13.3	15.6	10.5	9.9	11.8	11.7	12.5
20–29	23.4	24.0	28.6	24.3	23.2	21.2	22.2	25.2	22.1	20.4
30–39	19.2	19.1	21.4	18.8	16.2	17.6	18.7	19.4	17.8	17.6
40–49	13.7	13.7	12.3	12.8	10.0	14.6	14.9	13.8	14.1	13.8
50–59	9.1	9.9	6.5	8.4	6.6	10.4	11.1	8.8	10.1	9.2
60+	9.1	10.8	7.1	8.0	9.2	15.3	14.1	11.0	12.1	13.3
**Total (n)**	**100.0 (1,505,492)**	**100.0 (390,747)**	**100.0 (81,798)**	**100.0 (4,210,557)**	**100.0 (102,262)**	**100.0 (6,768,722)**	**100.0 (2,151,841)**	**100.0 (310,191)**	**100.0 (13,501,871)**	**100.0 (84,819)**

North and Northeast, Brazil, 2010.

Table 1 also shows that among all studied groups in both regions, Indigenous women had the lowest levels of education. In the North and Northeast, 82.4% and 64.3%, respectively, were illiterate or had only incomplete primary education. Black and brown women occupied intermediate positions in terms of schooling, while yellow and white women presented higher levels of formal education. Indigenous women also tended to be younger, with higher concentrations in the first two age brackets: 10–14 and 15–19 years (Table 1).

In both of the studied regions, just over half of census interviewees answered census questions directly (51.2% in the North and 54.4% in the Northeast) (Table 2). In other words, 45.6% to 48.8% of the interviews were carried out with proxy respondents, either household co-residents or non-residents. The percentage of females who answered the census questions directly was somewhat higher in rural areas, especially in the North (55.6%). Older women, especially those aged between 50 and 59 years, were more likely to respond directly to interviewers. The relationship between type of respondent and women´s schooling did not follow a clear pattern. Indigenous women in the North region had the highest percentage of interviews carried out with a non-resident (12.7% total; 15.0% and 3.0% in rural and urban areas, respectively). It is remarkable that the other color/race groups in the North region had comparatively very low frequencies of interviews carried out with non-residents: white – 4.0%, black – 2.9%, yellow – 2.5%, and brown – 3.0%. Compared to the North region, Indigenous and non-Indigenous women in the Northeast presented much smaller differences in patterns of response according to type of respondent.

**Table 2:** Type of respondent of the 2010 Brazilian census, according to urban/rural status, educational attainment, color/race, and age, stratified by region.

Variables	Type of respondent					
	North			Northeast		
	Woman directly	Co-resident	Non-resident	Woman directly	Co-resident	Non-resident
**Urban/rural status**						
Urban	49.9	46.8	3.3	53.4	43.0	3.6
Rural	55.6	40.8	3.6	57.6	39.3	3.1
**Educational attainment**						
Illiterate/incomplete primary education	50.1	46.5	3.4	55.4	41.1	3.5
Complete primary education/ incomplete secondary education	52.2	45.0	2.8	52.8	44.3	2.9
Complete secondary education/ incomplete high school	53.1	43.2	3.7	53.5	42.8	3.7
Complete high school	52.3	43.7	4.0	54.3	42.1	3.6
Unknown	36.4	61.4	2.2	35.8	61.2	3.0
**Color/race**						
White	45.4	50.6	4.0	49.6	46.3	4.1
Black	57.3	39.8	2.9	59.3	37.4	3.3
Yellow	62.1	35.4	2.5	63.4	34.3	2.3
Brown	52.7	44.3	3.0	55.9	40.9	3.2
Indigenous	41.9	45.4	12.7	55.2	41.0	3.8
**Age (years)**						
10–14	16.9	80.4	2.7	17.5	80.2	2.3
15–19	34.4	63.0	2.6	34.5	63.2	2.3
20–29	55.4	40.9	3.7	54.5	41.9	3.6
30–39	62.8	33.8	3.4	64.9	31.8	3.4
40–49	63.6	33.4	3.0	66.2	30.8	3.0
50–59	65.2	31.2	3.6	69.0	27.6	3.4
60+	60.8	33.7	5.5	64.6	29.5	5.9
**Total**	**51.2**	**45.4**	**3.4**	54**.4**	**42.1**	**3.5**

North and Northeast, Brazil, 2010.

Table 3 shows parity means according to urban/rural status, education, color/race, age and type of respondent, in the North and Northeast. This mean count is slightly higher in the Northeast (2.15 children) compared to the North (2.08 children). Parity was higher in rural areas, among groups with lower educational attainment, and, as expected, among older women. Indigenous respondents showed the highest mean parity, followed by black women. White and yellow women presented the lowest parities. Regardless of color/race, parity means were considerably higher when the respondent answered interview questions about herself – such values were 93.5%-101.4% higher when compared to answers given by a co-resident and 15.6%-21.7% higher than those given by a non-resident.

**Table 3:** Mean count of children (parity), reported to the 2010 Brazilian census, according to urban/rural status, educational attainment, color/race, age and type of respondent, stratified by region.

Variables	Mean (95%CI)	
	North	Northeast
**Urban/rural status**		
Urban	1.95 (1.94 – 1.96)	1.98 (1.98 – 1.99)
Rural	2.54 (2.53 – 2.56)	2.65 (2.64 – 2.66)
**Educational attainment**		
Illiterate/incomplete primary education	2.65 (2.64 – 2.66)	2.90 (2.89 – 2.90)
Complete primary education/ incomplete secondary education	1.53 (1.52 – 1.54)	1.32 (1.31 – 1.32)
Complete secondary education/ incomplete high school	1.43 (1.42 – 1.44)	1.15 (1.14 – 1.15)
Complete high school	1.39 (1.38 – 1.41)	1.26 (1.25 – 1.27)
Unknown	0.69 (0.64 – 0.74)	0.53 (0.51 – 0.55)
**Color/race**		
White	1.90 (1.89 – 1.92)*	2.02 (2.01 – 2.02) *
Black	2.38 (2.35 – 2.41)	2.28 (2.27 – 2.29)
Yellow	1.87 (1.81 – 1.92)*	2.04 (2.01 – 2.07)*
Brown	2.12 (2.11 – 2.13)	2.19 (2.19 – 2.20)
Indigenous	2.46 (2.40 – 2.51)	2.52 (2.45 – 2.58)
**Age (years)**		
10–14	0.01 (0.01 – 0.01)	0.01 (0.01 – 0.01)
15–19	0.23 (0.22 – 0.23)	0.16 (0.16 – 0.17)
20–29	1.26 (1.25 – 1.26)	0.97 (0.97 – 0.97)
30–39	2.42 (2.41 – 2.43)	2.03 (2.03 – 2.04)
40–49	3.23 (3.22 – 3.25)	2.82 (2.81 – 2.83)
50–59	4.25 (4.22 – 4.28)	3.91 (3.89 – 3.92)
60+	5.84 (5.80 – 5.87)	5.67 (5.65 – 5.68)
**Type of respondent**		
Woman directly	2.69 (2.68 – 2.70)	2.74 (2.74 – 2.75)
Co-resident	1.39 (1.38 – 1.40)	1.36 (1.35 – 1.36)
Non-resident	2.21 (2.16 – 2.25)	2.37 (2.34 – 2.39)
**Total**	**2.08 (2.08 – 2.09)**	**2.15 (2.14 – 2.15)**

North and Northeast, Brazil, 2010.

*These are mean counts, which are not statistically significantly different from each other within the North or the Northeast regions (p > 0.05, according to the Wald test of heterogeneity). Obs.1: 95%CI = 95% confidence interval. Obs.2: The mean parity is statistically significantly different (p < 0.05, according to the Wald test of heterogeneity) when the North and Northeast regions are compared.

When parity was estimated according to all covariates in ZINB regression models, Indigenous women showed values 18%-39% higher when compared to white women (Table 4). Considering all color/race categories combined and comparing parity means by type of respondent, with the self-reported category as reference, the values were lower in the case of co-resident respondents, varying from 1.0% (rural North) to 7.0% (urban Northeast) less. The interaction between color/race and type of respondent revealed a central result of the present analysis: Indigenous women from the rural North whose census questions were answered by a non-resident showed 16.0% (0.84, 95% CI 0.75 – 0.93) fewer children when compared to white women who provided their own information. This was the largest measure of effect detected among all color/race groups and regions. Although the values for Indigenous and yellow women in the urban North were even lower (0.78 and 0.82, respectively), they were not statistically significant. Concerning parity for Indigenous women in the North in comparison to white women directly responding to census interviewers, the values were 10.0% (0.90, 95% CI 0.82 – 0.99) and 7.0% (0.93, 95% CI 0.88 – 0.99) lower in urban and rural settings, respectively, when the answers were provided by a co-resident. There was a negative association between parity and education, as well as a positive relation with age. Number of household rooms and number of household members were not associated with parity.

**Table 4:** Zero-inflated Negative Binomial regression to estimate the relation between color/race and parity, stratified by region and urban/rural status, adjusted for age, educational attainment, number of household rooms and household members.

Variables	Regression coefficients for parity (95% confidence interval)			
	North		Northeast	
	Urban	Rural	Urban	Rural
**Color/race**				
White	1.00 (-)	1.00 (-)	1.00 (-)	1.00 (-)
Black	1.10 (1.08 – 1.12) ^¶^	1.15 (1.12 – 1.19) ^¶^	1.04 (1.03 – 1.05) ^¶^	1.09 (1.07 – 1.10) ^¶^
Yellow	1.08 (1.04 – 1.12) ^¶^	1.03 (0.96 – 1.11)	1.06 (1.04 – 1.08) ^¶^	1.09 (1.06 – 1.12) ^¶^
Brown	1.12 (1.11 – 1.14) ^¶^	1.18 (1.16 – 1.20) ^¶^	1.09 (1.08 – 1.09) ^¶^	1.09 (1.08 – 1.10) ^¶^
Indigenous	1.39 (1.31 – 1.47) ^¶^	1.34 (1.29 – 1.39) ^¶^	1.18 (1.13 – 1.24) ^¶^	1.27 (1.22 – 1.32) ^¶^
**Type of respondent**				
Woman directly	1.00 (-)	1.00 (-)	1.00 (-)	1.00 (-)
Co-resident	0.98 (0.96 – 0.99) ^¶^	0.99 (0.97 – 1.03)	0.93 (0.92 – 0.93) ^¶^	0.94 (0.92 – 0.95) ^¶^
Non-resident	0.97 (0.93 – 1.01)	1.04 (0.96 – 1.12)	0.97 (0.95 – 0.98) ^¶^	0.98 (0.95 – 1.01)
**Color/race X type of respondent (interaction term)** *				
Black; co-resident	0.94 (0.91 – 0.98) ^¶^	0.97 (0.92 – 1.02)	0.95 (0.94 – 0.97) ^¶^	0.98 (0.95 – 1.01)
Black; non-resident	1.01 (0.91 – 1.12)	1.00 (0.86 – 1.18)	0.92 (0.88 – 0.96) ^¶^	0.88 (0.83 – 0.94) ^¶^
Yellow; co-resident	0.90 (0.83 – 0.97) ^¶^	0.94 (0.83 – 1.07)	0.96 (0.92 – 1.00)	0.97 (0.92 – 1.04)
Yellow; non-resident	0.78 (0.59 – 1.04)	0.98 (0.77 – 1.26)	0.90 (0.80 – 1.01)	1.02 (0.88 – 1.18)
Brown; co-resident	0.93 (0.91 – 0.95) ^¶^	0.93 (0.90 – 0.96) ^¶^	0.93 (0.92 – 0.94) ^¶^	0.96 (0.95 – 0.98) ^¶^
Brown; non-resident	0.96 (0.92 – 1.01)	0.90 (0.83 – 0.98) ^¶^	0.90 (0.88 – 0.93) ^¶^	0.91 (0.88 – 0.95) ^¶^
Indigenous; co-resident	0.90 (0.82 – 0.99) ^¶^	0.93 (0.88 – 0.99) ^¶^	0.96 (0.88 – 1.04)	0.98 (0.91 – 1.06)
Indigenous; non-resident	0.82 (0.63 – 1.07)	0.84 (0.75 – 0.93) ^¶^	0.99 (0.84 – 1.17)	0.91 (0.78 – 1.06)
**Educational attainment**				
Illiterate/incomplete primary education	1.00 (-)	1.00 (-)	1.00 (-)	1.00 (-)
Complete primary education/ incomplete secondary education	0.74 (0.73 – 0.75) ^¶^	0.75 (0.74 – 0.77) ^¶^	0.72 (0.71 – 0.72) ^¶^	0.72 (0.72 – 0.73) ^¶^
Complete secondary education/ incomplete high school	0.56 (0.56 – 0.57) ^¶^	0.59 (0.58 – 0.61) ^¶^	0.53 (0.52 – 0.53) ^¶^	0.53 (0.53 – 0.54) ^¶^
Complete high school	0.45 (0.44 – 0.45) ^¶^	0.53 (0.51 – 0.56) ^¶^	0.44 (0.44 – 0.45) ^¶^	0.52 (0.50 – 0.53) ^¶^
Unknown	0.58 (0.52 – 0.63) ^¶^	0.79 (0.67 – 0.94) ^¶^	0.59 (0.56 – 0.62) ^¶^	0.69 (0.63 – 0.77) ^¶^
**Age (years)**				
10–14	0.16 (0.11 – 0.22) ^¶^	0.11 (0.07 – 0.16) ^¶^	0.17 (0.13 – 0.22) ^¶^	0.15 (0.11 – 0.19) ^¶^
15–19	0.17 (0.16 – 0.18) ^¶^	0.18 (0.17 – 0.19) ^¶^	0.18 (0.17 – 0.18) ^¶^	0.18 (0.17 – 0.18) ^¶^
20–29	0.61 (0.61 – 0.62) ^¶^	0.60 (0.59 – 0.61) ^¶^	0.62 (0.61 – 0.62) ^¶^	0.60 (0.59 – 0.60) ^¶^
30–39	1.00 (-)	1.00 (-)	1.00 (-)	1.00 (-)
40–49	1.26 (1.24 – 1.27) ^¶^	1.29 (1.27 – 1.32) ^¶^	1.28 (1.27 – 1.29) ^¶^	1.40 (1.38 – 1.41) ^¶^
50–59	1.58 (1.56 – 1.60) ^¶^	1.61 (1.58 – 1.64) ^¶^	1.72 (1.70 – 1.73) ^¶^	1.92 (1.91 – 1.94) ^¶^
60+	2.13 (2.10 – 2.16) ^¶^	1.91 (1.87 – 1.94) ^¶^	2.54 (2.52 – 2.56) ^¶^	2.50 (2.47 – 2.52) ^¶^
**Number of rooms in the household**				
1+	1.00 (1.00 – 1.00)	1.00 (1.00 – 1.00)	1.00 (1.00 – 1.00) ^¶^	1.00 (1.00 – 1.00)
**Number of household members**				
1+	1.00 (1.00 – 1.00)	1.00 (1.00 – 1.00)	1.00 (1.00 – 1.00)	1.00 (1.00 – 1.00)

2010 Brazilian census, North and Northeast, 2010.

*The reference category was white women who directly responded to census takers.

^¶^Statistically significant associations (p < 0.05), according to the Wald test of heterogeneity. Obs.: Only coefficients (which are incidence rate ratios) for the count of children born to the studied women are presented in this table – coefficients estimated by the logistic part of the model are presented as supporting information (see S1 Table).

**Fig 1:**
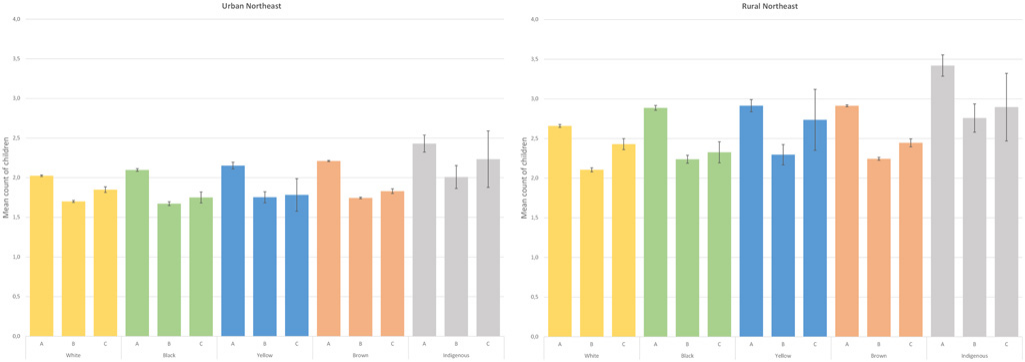
Parity (and its respective 95% confidence intervals) according to color/race and type of respondent, adjusted for age, educational attainment, number of household rooms and number of household members. 2010 Brazilian census, Northeast, Brazil, 2010. Obs: A = information provided directly by the woman, B = information provided by a co-resident, C = information provided by a non-resident.

**Fig 2:**
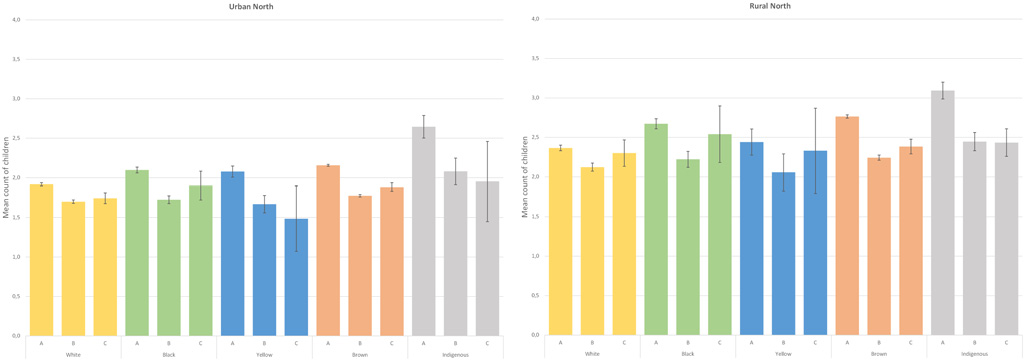
Parity (and its respective 95% confidence intervals) according to color/race and type of respondent, adjusted for age, educational attainment, number of household rooms and number of household members. 2010 Brazilian census, North, Brazil, 2010. Obs: A = information provided directly by the woman, B = information provided by a co-resident, C = information provided by a non-resident.

Model-predicted estimates, shown in Figs 1 and 2, demonstrate that parity is affected by type of respondent in all color/race categories in both urban and rural areas of the two regions investigated. In general, type of respondent has a relatively similar impact on all color/race groups in the Northeast, most often with the lowest parity means associated with co-resident respondents and the highest values when the information was reported directly (Fig 1). In most instances, means did not differ when the information was provided by a co-resident or by a non-resident, as the confidence intervals overlap. In comparative terms, the impact of interviews carried out with proxy respondents was considerably higher among Indigenous women from the North, especially in the rural area (Fig 2). While the mean count of self-reported parity was 3.1 (95% CI 3.0 – 3.2) in the rural North, the values were similar (co-resident: 2.4, 95% CI 2.3 – 2.6; non-resident: 2.4, 95% CI 2.3 – 2.6) when another household member or a non-resident provided the answer. In the urban North, parity means were just slightly higher when provided directly by the women (2.6, 95% CI 2.5 – 2.8) in comparison to the situation in which the information was given by a co-resident (2.1, 95% CI 1.9 – 2.2) or a non-resident (2.0, 95% CI 1.4 – 2.5).

## Discussion

In the small yet growing literature on the demography of Indigenous populations in Brazil based on national census data [12–15, 23, 24], little attention has been given to the fact that these culturally differentiated societies might present characteristics, including language affiliation, that could influence patterns of responses to census interviewers. The results of this study show that Indigenous women, especially those from the North region, respond directly to census interviewers in a much lower percentage compared to women of other color/race categories. A remarkable finding is that 15.9% of Indigenous women from the North region had their information provided by non-household residents, which is a frequency well-above that of women of other color/race categories in the same region, which ranged from 2.5% (yellow) to 4.0% (white). It is also revealing that, in the case of Indigenous women from the Northeast region, no such differences were found.

Results of this investigation also revealed that there are major differences in the mean parity depending on who responded to the census interviewers. When data are not directly provided by the woman, but by proxy respondents (either a co-resident or a non-resident), mean values of parity tend to be much lower. This is not only the case for Indigenous women, but applies to all color/race groups in the two investigated regions, both in urban and rural areas. The higher percentage of Indigenous women whose information was provided by a non-resident in the North (12.7%) may indicate that the relation between parity and type of respondent could be different in the two geographic regions studied. However, after controlling for a number of covariates through multivariable analyses, it was found that underreporting of Indigenous women´s parity by co-residents and non-residents was similar in the North and Northeast. It should be mentioned, though, that comparisons of parity estimates taking into consideration urban/rural status and type of respondent indicate that parity underreporting was particularly strong in Indigenous women living in the rural North (16% less in comparison to White women who responded directly to census interviewers).

Regardless of the woman´s color/race, the underreporting of parity possibly stems from the greater likelihood that proxy respondents would not be able to provide as complete information on number of children ever had, as would also be the case for many other variables related to the women’s reproductive trajectory. For instance, proxy respondents may not have knowledge of a woman’s deceased children and they might not know of children who do not live with their parents in the same household. When the answer is not provided directly by the woman, these children may not be taken into account. The fact that Indigenous populations in Brazil present higher infant mortality rates might exert additional influence on the differences between proxy- and mother-reported parities, which were larger among Indigenous women in comparison to those of other colors/races [12, 25]. The combination of these two dimensions – lower frequencies of Indigenous women directly responding to census interviewers and less complete data provided by other respondents – leads to a synergetic effect that helps to underestimate even more parity for Indigenous women, especially in the North region.

Which factors could explain the pattern of lower direct response to census interviewers by Indigenous women from the North region? It is very likely that socio-cultural aspects play a leading role. Compared to other Brazilian regions, Indigenous societies that live in the North have been able not only to preserve more their native languages, but also their traditional territories [21, 22]. The reason behind this is historical. In Brazil, the North region was the last one to experience large-scale colonization, particularly from the 1960s onward [26]. To a certain extent, despite the very high rates of mortality and violence, in more recent times, Indigenous movements have been able to assure some rights to Indigenous societies from the North that were not available to those in other parts of the country. Colonization of the Northeast by non-Indians took place starting in the sixteenth century, which meant that Indigenous societies living in this region were the first to experience the devastating impacts of the arrival of Europeans, resulting in massive depopulation and loss of territories and languages [21, 22, 27]. Data from the Brazilian 2010 census show how some of the cultural and socioeconomic characteristics of Indigenous societies at present in Brazil reflect the long-lasting effects of five hundred years of colonization by non-Indians. Whereas 26.2% of those self-declared Indigenous >5 years of age in the North region did not speak Portuguese, but only Indigenous languages, the frequency in the Northeast was only 3.2% [13]. Having this complex historical and socio-political scenario as background, the fact that the Brazilian national census is carried out in Portuguese might influence patterns of data collection in instances, such as those of Indigenous groups, in which part or the totality of the population under investigation does not use Portuguese in their day-to-day communication.

In the context of this paper, the variable concerning who provided the information for census interviewers has been a central one. A relevant issue is the identity of the non-residents that, as we have seen for the North region in particular, played an important role in providing data on Indigenous women to the census interviewers. According to the IBGE, especially in Brazilian urban centers, a non-resident respondent is often a paid-by-the-day domestic worker who happens to be the only person available at the household at the time of the visit of the census interviewers [19]. For Indigenous populations, it is unlikely that non-resident respondents are domestic workers, as they are not common in Indigenous communities. Instead, it is more likely that, in the case of Indigenous women, non-resident respondents include health and education professionals, who work and live in the communities, many of them of Indigenous ancestry themselves. Other nationally representative investigations, such as the recent First National Survey on Indigenous Nutrition and Health, carried out in 2008 [28], have reported the important role played by health and education professionals in the dynamics of developing a rapport with the community, to the extent of helping in the translation of the interviews. Even if these professionals live and work in indigenous communities, and are therefore familiar with local cultural characteristics and perhaps language, they are not necessarily acquainted with each woman’s individual reproductive history to the point that they can provide parity information with the level of accuracy expected from firsthand accounts.

Although this paper has focused on a specific set of variables related to parity, the issues raised might have far-reaching implications. Just as the quantification of parity of Indigenous women might be influenced by the combination of lower rates of self-response and lower estimates due to increased proportion of responses by other residents and non-residents, the values of several other demographic variables, ranging from mortality estimates to socioeconomic conditions, might also be affected. Closely related to parity, fertility estimates are a good case in point. Analysis based on the Brazilian 2010 census data [29] has shown high total fertility rates (TFR) for Indigenous women in Brazil: 2.90 in the Southeast; 3.01 in the Northeast; 3.74 in the South; 4.30 in the Center-West; and 4.92 in the North, totaling 3.88 for the country as a whole [13]. As has been pointed out in several investigations aimed at characterizing patterns of fertility transition in Brazil [30–32], these inter-regional differences in Indigenous fertility possibly also derive from the interaction of a complex set of behavioral and socio-economic factors, including education, women’s participation in the labor market, access to contraceptives, among others. It might be argued that if the proportion of this class of respondents were taken into consideration, the TFR values for Indigenous women living in the North would be even higher and thereby affect the magnitudes of inter-regional fertility differentials for this population segment. On a broader scale, analyses of demographic patterns of Brazilian populations (Indigenous and non-Indigenous), including the important issues of mortality and fertility transitions, should take into consideration that values of demographic indicators could be affected by aspects that do not strictly stem from demographic determinants.

## Conclusions

Analyses of the demographic characteristics of Indigenous populations based on national census data are becoming increasingly important as elements in the debates on health, education, and other socioeconomic inequalities worldwide [1–3, 33–35]. However, as Axelsson and Sköld [1] have remarked on the limitations of large-scale surveys not specifically aimed at collecting data on Indigenous peoples, “externally produced censuses, surveys and administrative data are often inaccurate, when it comes to representing Indigenous social structures.” At the same time that it is fundamental to expand the collection of demographic data on Indigenous populations, it should not be overlooked that data quality might be affected by the specific conditions under which data collection takes place. The case study on Indigenous peoples from Brazil presented in this paper provides compelling evidence that Indigenous women present patterns of response to census interviewers that, ultimately, influence the quantification of demographic outcomes. This was also observed for non-Indigenous women, but not to the same degree.

Some recent initiatives in Latin America, such as the inclusion in the census forms of questions on Indigenous languages, are welcome developments that might result in a much better characterization of Indigenous demography [10, 11, 36]. As long as specific routines for the collection of demographic data in culturally differentiated populations, such as Indigenous peoples, are not more broadly implemented at national levels, in Latin America and elsewhere, more attention should be paid to demographic differentials resulting from processes of data collection. It is needless to emphasize that these differentials might influence the definition and implementation of health public policies aimed at ameliorating the high degree of socioeconomic marginalization that characterize indigenous populations all over the world. The implementation of certain methodological alternatives in the Brazilian national censuses, such as the selection and training of census takers to work specifically in Indigenous territories, who preferably could be Indigenous individuals fluent in native languages and acquainted with native cultures, might be a productive means to improve data collection. Considering that parity differences according to type of respondent were observed in women of all color/race categories, further investigation of parity estimates for all regions of the country should be undertaken.

## Supporting Information

S1 Information Comparing and selecting the appropriate statistical model. (DOCX)

S2 Information Parameters of fit for regression models with and without the interaction term between color/race and type of informant. (DOCX)

S1 Table Zero-inflated Negative Binomial regression to estimate the relation between color/race and parity, stratified by region and urban/rural status, adjusted for age, educational attainment, number of household rooms and household members. 2010 Brazilian census, North and Northeast, 2010.
(DOCX)
