# Supplementary material for: Parity of Indigenous and Non-Indigenous Women in Brazil: Does the Reported Number of Children Born Depend upon Who Answers National Census Questions?
Source: PLoS One. 2015 Apr 14;10(4):e0123826. doi: 10.1371/journal.pone.0123826 (PMC4397089; doi:10.1371/journal.pone.0123826)
Supplement: S1 Information — (DOCX) [file pone.0123826.s001.docx]

**Comparing and selecting the appropriate statistical model**

ZINBs (Zero-inflated Negative Binomial) and related regression models are especially devised to analyze count data showing simultaneously an excess of zeroes and overdispersion.

The figure below shows that Poisson distribution underestimates the actual frequency of zeroes: while 21.1% of women were predicted to have no children by the Poisson model, the dataset actually had 38.3% women in this category. On the other hand, the ZINB (blue line) and the Zero-inflated Poisson (ZIP – green line) models predicted that 38.7% and 38.5% of women, respectively, would have no children, values closer to our observed frequency.

**S1 Supporting information. Observed probability for the count of children born to women (parity), and the corresponding predicted probabilities according to the Poisson, Zero-inflated Poisson, Negative Binomial, and Zero-inflated Negative Binomial models.**

Following recommendations set forth by Long and Freese [1], Poisson, Negative Binomial, ZIP, and ZINB models were also compared according to the Bayesian Information Criterion (BIC), Akaike Information Criterion (AIC), Likelihood Ratio Test, and Vuong Test in order to select the most appropriate regression model. The results of these comparisons are displayed in the table below.

**S1 Supporting information. Comparisons among distinct count regressions according to the Bayesian Information Criterion, Akaike Information Criterion, Likelihood Ratio Test, and Vuong Test in order to choose the best fitting model for the observed parity distribution.**

| Models being compared | Differences according to the Bayesian Information Criterion (BIC)  and the Akaike Information Criterion (AIC) | | Likelihood Ratio Test | Vuong Test |
| --- | --- | --- | --- | --- |
| Poisson  vs.  Negative binomial | BIC _Poisson_ – BIC _Negative Binomial_ =  –1.770 – (–1.859) = 0,089 | AIC _Poisson_ – AIC _Negative Binomial_ =  4.260 – 3.753 = 0.507 | <0.001 | - |
| Poisson  vs.  Zero-inflated Poisson | BIC _Poisson_ – BIC _Zero-inflated Poisson_ =  –1.770 – (–1.868) = 0,098 | AIC _Poisson_ – AIC _Zero-inflated Poisson_ =  4.260 – 3.701 = 0.560 | - | <0.001 |
| Poisson  vs.  Zero-inflated Negative Binomial | BIC _Poisson_ – BIC _Zero-inflated Negative Binomial_ =  –1.770 – (–1.885) = 0,115 | AIC _Poisson_ – AIC _Zero-inflated Negative Binomial_ =  4.260 – 3.603 = 0.657 | - | - |
| Negative Binomial  vs.  Zero-inflated Poisson | BIC _Negative Binomial_ – BIC _Zero-inflated Poisson_ =  –1.859 – (–1.868) = 0,009 | AIC _Negative Binomial_ – AIC _Zero-inflated Poisson_ =  3.753 – 3.701 = 0.052 | - | - |
| Negative Binomial  vs.  Zero-inflated Negative Binomial | BIC _Negative Binomial_ – BIC _Zero-inflated Poisson_ =  –1.859 – (–1.885) = 0,026 | AIC _Negative Binomial_ – AIC _Zero-inflated Poisson_ =  3.753 – 3.603 = 0.150 | - | <0.001 |
| Zero-inflated Poisson  vs.  Zero-inflated Negative Binomial | BIC _Zero-inflated Poisson_ – BIC _Zero-inflated Negative Binomial_ =  –1.868 – (–1.885) = 0,017 | AIC _Zero-inflated Poisson_ – AIC _Zero-inflated Negative Binomial_ =  3.701 – 3.603 = 0.098 | <0.001 | - |

Essentially, the table above shows that any model assuming overdispersion is more appropriate – e.g. the Negative Binomial regression fits better than the Poisson model. This table also demonstrates that zero-inflated models show superior results. When the ZIP and the ZINB models are compared against each other, the results indicate that the latter regression should be adopted (cf. the last table row). Another reason for using the ZINB model is that the parity distribution is overdispersed, i.e. the variance is much higher than the mean. In the case of the present dataset, the global mean parity was 2.13, and the corresponding variance, 8.01, clearly indicating that the ZINB model is the best to analyze the data.

**References**

1. Long JS, Freese J. Regression models for categorical dependent variables using Stata. Texas: Stata Press; 2006.
