# Supplementary material for: Parity of Indigenous and Non-Indigenous Women in Brazil: Does the Reported Number of Children Born Depend upon Who Answers National Census Questions?
Source: PLoS One. 2015 Apr 14;10(4):e0123826. doi: 10.1371/journal.pone.0123826 (PMC4397089; doi:10.1371/journal.pone.0123826)
Supplement: S2 Information — (DOCX) [file pone.0123826.s002.docx]

**Model diagnostics**

The table below shows fit statistics for the regression models with and without interaction terms.

**S2 Supporting information. Parameters of fit for regression models with and without the interaction term between color/race and type of informant.**

| Parameter of fit | Geographic region and urban/rural status | | | | | | | |
| --- | --- | --- | --- | --- | --- | --- | --- | --- |
|  | Urban North | | Rural North | | Urban Northeast | | Rural Northeast | |
|  | Model ***without*** interaction term | Model ***with*** interaction term | Model ***without*** interaction term | Model ***with*** interaction term | Model ***without*** interaction term | Model ***with*** interaction term | Model ***without*** interaction term | Model ***with*** interaction term |
| Likelihood ratio test comparing models | <0.001 | | <0.001 | | <0.001 | | <0.001 | |
| Bayesian Information Criterion | 7406455.3 | 7405676.3 | 2446512.7 | 2446284.1 | 27190000 | 27180000 | 9584118.0 | 9583775.8 |
| Akaike Information Criterion | 7406048.1 | 7405185.6 | 2446139.9 | 2445834.9 | 27180000 | 27180000 | 9583688.0 | 9583257.6 |
| Overall interpretation | There is support for the model ***with*** the interaction term | | There is support for the model ***with*** the interaction term | | There is support for the model ***with*** the interaction term | | There is support for the model ***with*** the interaction term | |

With no exception, all parameters of fit indicate that the models including the interaction term perform better.
