# Supplementary material for: Parity of Indigenous and Non-Indigenous Women in Brazil: Does the Reported Number of Children Born Depend upon Who Answers National Census Questions?
Source: PLoS One. 2015 Apr 14;10(4):e0123826. doi: 10.1371/journal.pone.0123826 (PMC4397089; doi:10.1371/journal.pone.0123826)
Supplement: S1 Table — 2010 Brazilian census, North and Northeast, 2010. (DOCX) [file pone.0123826.s003.docx]

**S1 Table. Zero-inflated Negative Binomial regression to estimate the relation between color/race and parity, stratified by region and urban/rural status, adjusted for age, educational attainment, number of household rooms and household members. 2010 Brazilian census, North and Northeast, 2010.**

| **Variable** | **Regression coefficients for parity (95% confidence interval)** | | | |
| --- | --- | --- | --- | --- |
|  | **North** | | **Northeast** | |
|  | **Urban** | **Rural** | **Urban** | **Rural** |
| Color/race |  |  |  |  |
| White | 0.00 (-) | 0.00 (-) | 0.00 (-) | 0.00 (-) |
| Black | 0.19 (0.06 – 0.32) ^¶^ | 0.42 (0.26 – 0.58) ^¶^ | 0.05 (-0.01 – 0.11) | 0.00 (-0.07 – 0.07) |
| Yellow | -0.10 (-0.37 – 0.16) | 0.05 (-0.41 – 0.50) | -0.14 (-0.29 – 0.00) | -0.17 (-0.33 – -0.00) ^¶^ |
| Brown | 0.02 (-0.05 – 0.09) | 0.25 (0.15 – 0.35) ^¶^ | -0.09 (-0.13 – -0.06) ^¶^ | -0.08 (-0.12 – -0.04) ^¶^ |
| Indigenous | 0.17 (-0.22 – 0.56) | 0.51 (0.35 – 0.67) ^¶^ | -0.35 (-0.64 – -0.05) ^¶^ | -0.31 (-0.53 – -0.10) ^¶^ |
| Type of respondent |  |  |  |  |
| Woman directly | 0.00 (-) | 0.00 (-) | 0.00 (-) | 0.00 (-) |
| Co-resident | 1.53 (1.45 – 1.61) ^¶^ | 1.37 (1.29 – 1.45) ^¶^ | 1.27 (1.23 – 1.31) ^¶^ | 1.53 (1.49 – 1.57) ^¶^ |
| Non-resident | 1.12 (0.95 – 1.29) ^¶^ | 0.99 (0.82 – 1.16) ^¶^ | 0.85 (0.77 – 0.93) ^¶^ | 0.88 (0.79 – 0.97) ^¶^ |
| Educational attainment |  |  |  |  |
| Illiterate/incomplete primary education | 0.00 (-) | 0.00 (-) | 0.00 (-) | 0.00 (-) |
| Complete primary education/ incomplete secondary education | 0.24 (0.15 – 0.32) ^¶^ | 0.07 (-0.03 – 0.17) | 0.19 (0.14 – 0.24) ^¶^ | 0.38 (0.33 – 0.43) ^¶^ |
| Complete secondary education/ incomplete high school | 0.65 (0.56 – 0.75) ^¶^ | 0.19 (0.05 – 0.33) ^¶^ | 0.83 (0.78 – 0.88) ^¶^ | 0.84 (0.78 – 0.90) ^¶^ |
| Complete high school | 1.11 (0.97 – 1.26) ^¶^ | 0.17 (-0.21 – 0.56) ^¶^ | 1.19 (1.10 – 1.27) ^¶^ | 0.58 (0.41 – 0.75) ^¶^ |
| Unknown | 0.85 (0.49 – 1.22) ^¶^ | 0.93 (0.61 – 1.25) ^¶^ | 0.99 (0.78 – 1.20) ^¶^ | 1.48 (1.24 – 1.71) ^¶^ |
| Age (years) |  |  |  |  |
| 10-14 | 7.90 (7.50 – 8.29) ^¶^ | 5.94 (5.55 – 6.33) ^¶^ | 8.64 (8.32 – 8.97) ^¶^ | 7.11 (6.84 – 7.38) ^¶^ |
| 15-19 | 3.92 (3.69 – 4.16) ^¶^ | 2.39 (2.25 – 2.54) ^¶^ | 4.56 (4.33 – 4.78) ^¶^ | 3.21 (3.11 – 3.30) ^¶^ |
| 20-29 | 2.12 (1.92 – 2.32) ^¶^ | 0.90 (0.78 – 1.03) ^¶^ | 2.73 (2.52 – 2.94) ^¶^ | 1.35 (1.27 – 1.43) ^¶^ |
| 30-39 | 0.00 (-) | 0.00 (-) | 0.00 (-) | 0.00 (-) |
| 40-49 | -0.27 (-0.55 – 0.12) | 0.02 (-0.13 – 0.17) | -1.10 (-1.75 – -0.45) ^¶^ | 0.31 (0.21 – 0.41) ^¶^ |
| 50-59 | 0.77 (0.54 – 0.99) ^¶^ | 0.06 (-0.09 – 0.22) | 1.24 (1.00 – 1.47) ^¶^ | 0.83 (0.74 – 0.92) ^¶^ |
| 60+ | 1.49 (1.26 – 1.71) ^¶^ | 0.32 (0.18 – 0.46) ^¶^ | 2.41 (2.18 – 2.63) ^¶^ | 1.17 (1.08 – 1.25) ^¶^ |
| Number of rooms in the household |  |  |  |  |
| 1+ | -0.02 (-0.03 – -0.00) ^¶^ | -0.01 (-0.03 – 0.01) | 0.00 (-0.01 – 0.01) ^¶^ | 0.00 (-0.00 – 0.01) |
| Number of household members |  |  |  |  |
| 1+ | 0.01 (-0.00 – 0.03) | -0.00 (-0.02 – 0.02) | 0.00 (-0.00 – 0.01) | 0.00 (-0.00 – 0.01) |

^¶^Statistically significant associations (p < 0.05), according to the Wald test of heterogeneity. Obs.: Only coefficients (which are in the logarithmic scale) for the logistic part of the regression model are presented.
